# The contribution of super typhoons to tropical cyclone activity in response to ENSO

**DOI:** 10.1038/s41598-019-41561-y

**Published:** 2019-03-25

**Authors:** Nam-Young Kang, Dongjin Kim, James B. Elsner

**Affiliations:** 1 National Typhoon Center, Korea Meteorological Administration, Seoul, South Korea; 2 Department of Geography, Florida State University, Tallahassee, USA

**Keywords:** Atmospheric science, Natural hazards

## Abstract

Western North Pacific tropical cyclone (TC) activity is known to have a significant response to El Niño and Southern Oscillation (ENSO). Among the TC intensity classifications, “super typhoon”, is widely used as a symbolic word for warning people about the potential for experiencing the most severe typhoon. This study quantifies the contribution of super typhoons to TC activity in response to ENSO, where the Southern Oscillation Index (SOI) is used to indicate the internal variation of ENSO. It is found that the number and the genesis location of non-super typhoons are little influenced by ENSO, and the changes are mostly by the addition of super typhoons. Historical levels of El Niño and La Niña span from −2σ to +2σ of the SOI. Over this range of SOI values, the mean response is 5.6 super typhoons (7.1 for El Niño and 1.5 for La Niña) which nearly matches the mean response overall of 6 storms (24.6 for El Niño and 18.6 for La Niña). The spatial distribution of TC genesis locations for different ENSO conditions does not completely explain these results.

## Introduction

The El Niño and Southern Oscillation (ENSO) is one of the most influential climate variability patterns of global ocean and atmosphere, and there have been a number of investigations on its connection to tropical cyclones (TCs). TC activity in the western North Pacific (WNP) has been found to show significant response to ENSO by its vorticity, wind shear, and moist static energy^1^. In an El Niño environment, more TCs are likely to occur in the southeastern quadrant of the WNP^2,3^, which is the peripheral area of subtropical high modulated by ENSO. This suggests that longer-living and stronger TCs contribute to the larger TC acvitity^4–6^, which can be monitored by some activity indicators such as Accumulated Cyclone Energy (ACE)^7^ or Power Dissipation Index (PDI)^8^.

Since ACE and PDI represent merged factors such as storm count, intensity and lifetime and its deconvolution is not available, further understanding of each portion of the attributes has been limited. Recently, considerable progress has been made in the analysis framework for TC climate indicators. An empirical framework was designed to express a variability where intensity and frequency of TCs are combined through principal component analysis^9^. Within this framework, the contributions of intensity and frequency to TC activity can be individually identified.

Based on these recent works, this study tries to quantify the annual TC activity and the contribution of super typhoons in response to ENSO. The word “super typhoon” is reserved for the highest level of storm warning category by the U.S. Joint Typhoon Warning Center, and the word is widely used as symbolic for the strongest typhoons. The threshold lifetime-maximum intensities (LMIs) for TC and super typhoon are defined as 34 kt and 130 kt, respectively. Here, Southern Oscillation Index (SOI) is used to indicate ENSO status, and the annual proportion of super typhoons to indicate the annual intensity of WNP TCs. The study period is the 30 years from 1986 to 2015. Values for all variables are averaged over the period June to November.

## ENSO and the Southern Oscillation Index

Various indices have been employed to indicate the ENSO status in order understand its role in climate variability. For example, Niño 1 + 2, Niño 3, Niño 3.4 and Niño 4 are widely used indices, based on the sea surface temperature (SST) averaged over each various regions of the eastern and central tropical Pacific Ocean^10^. SOI is another metric of ENSO variability but using sea level pressure differences between Darwin and Tahiti. The response of tropical SST to global SST^11^ suggests that the SST-based metrics are likely to reflect localized global warming pattern. Even the highly contested so called “global warming hiatus”^12^ is not seen in global mean SST (GMSST)^13^.

**Figure 1:**
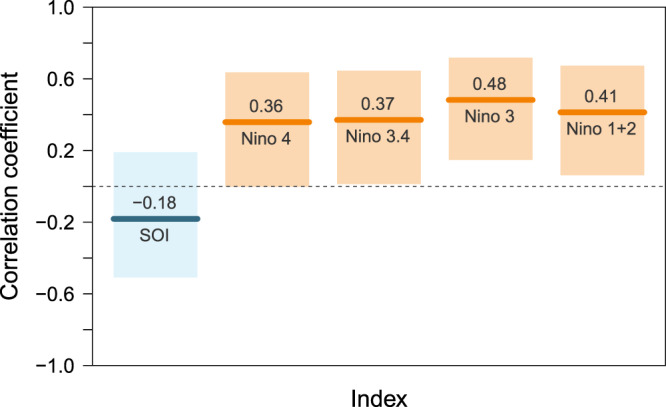
Correlation coefficients of ENSO (Niño) indices with GMSST. Line and shaded area represent correlation coefficient and the 95% confidence interval, respectively. SST-based indices such as Niño 4, Niño 3.4, Niño 3 and Niño 1 + 2 (in longitudinal order) are colored in orange.

On the other hand, SOI appears statistically and physically separate from global warming. Figure 1 shows the correlation of ENSO indices with GMSST, which represents the variation of global ocean warmth. Negative SOI values indicate the warm phase of ENSO, that is, El Niño. Importantly, SOI has little correlation with GMSST, while all the Niño indices are confirmed to have significantly positive correlation with global SST variation. As SST warming becomes distinct, the connection between SOI and GMSST by Bjerknes feedback^14^ might become muted. Thus for a better representation of an internal variation showing no forced trend, in this study SOI is used as the ENSO index.

## ENSO and Super Typhoons

**Figure 2:**
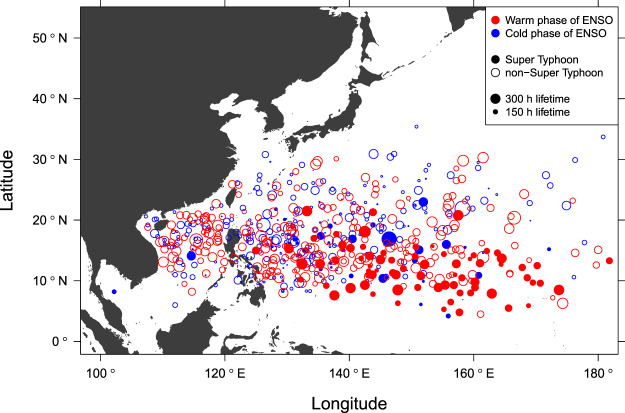
Spatial distribution of TC genesis locations and their LMIs. The genesis location in a warm phase (cold phase) of ENSO is marked by the red (blue) circle. The closed circle indicates a case with super typhoon intensity during its lifetime. Circle size corresponds to how long the TC existed (lifetime).

Figure 2 shows the spatial distribution of genesis locations of TCs. Size of the circle represents the length of time the storm existed as a TC. Among the TCs having longer lifetimes, super typhoons are more abundant across the south-eastern part of the area^3,4^. Here, the warm (cold) phase of ENSO is defined by a negative (positive) value of SOI. The map confirms that the number of super typhoons during the warm phase of ENSO is larger then the number during the cold phase. The occurrence of super typhoons to the east of 140°E and to the south of 20°N, is apparently greater in the warm phase of ENSO. It is noted on the other hand that the number and genesis location of non-super typhoons seem to be little influenced by ENSO (Supplementary Figs S1 and S2), and the changes are mostly by the addition of super typhoons. Thus the number of annual TCs in response to ENSO (Supplementary Fig. S3) might be simply the result of more super typhoons.

## Modeling TC Activity

**Figure 3:**
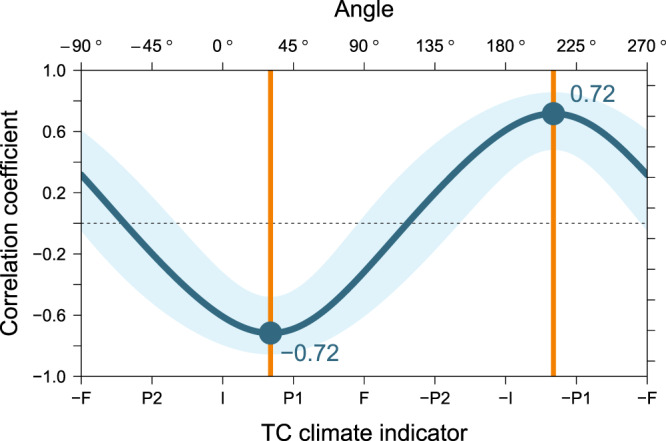
Correlation coefficients of TC climate indicators (TCIs) with SOI. Upper abscissa is labeled with angles corresponding to TCIs in the lower abscissa. Line and shaded area represent the correlation coefficient and each 95% confidence interval (CI), respectively. Values are shown at 0.1° intervals, and the highest correlation (+0.72 [0.48, 0.86] 95% CI) of TCI with El Niño (inverse sign of SOI) appears at 30.4° direction.

This study quantifies and explains the response of TC activity to ENSO. Annual TC activity can be indicated by a merged contribution of the TC climate attributes. Among others, ACE and PDI are well-known indicators of TC activity, where the TC count, intensity and lifetime are all involved. ACE and PDI reflect the role of stronger TCs by squared and cubed wind speeds, respectively, near the storm center, but the intensity factor in annual TC activity can be one giving a larger weight to the stronger TCs. For example, the portion of TCs over a certain threshold LMI can be an indicator of intensity on the annual time scale. In this study, the annual intensity (INT) is defined as the proportion of super typhoons among the total WNP TCs (Supplementary Fig. S4), while the annual frequency (FRQ) is defined as the number of total WNP TCs each year (Supplementary Fig. S5). As the number of super typhoons is divided by the total number of typhoons, the information on TC “amount” is removed from INT, and only the “proportion” remains. “Proportion” is considered independent of FRQ. INT and FRQ are used to form a two-dimensional space for TC climate variability^15^. Kang and Elsner^11^ suggest a TC climate indicator (TCI) as

1 $${\rm{TCI}}\equiv s({\rm{INT}})\cdot \,\cos \,{\rm{\theta }}+s({\rm{FRQ}})\cdot \,\sin \,{\rm{\theta }},$$

where *s*() returns standardized vector values, and where θ is the angle starting from INT, where the counterclockwise direction is positive. By Eq. (1), TCI is the projection of INT and FRQ on the directional variability at θ. TCI at 45° represents the principal component of the in-phase relationship (PC1) between INT and FRQ, while TCI at −45° (315°) their out-of-phase relationship (PC2). Figure 3 demonstrates the annual response of TCI to ENSO. PC2, INT, PC1, FRQ, and their negative vectors are denoted as P2, I, P1, F, -P2, -I, -P1 and -F, respectively. The strongest response of TCI to El Niño appears near PC1 direction at 30.4° (*r* = +0.72 [0.48, 0.86] 95% CI). For comparison, the correlations of ACE and PDI with -SOI (El Niño) are found 0.68 and 0.69 (Supplementary Fig. S6). This directional variability is denoted as TCI_opt_, which stands for ‘optimal’ TCI indicating the TC activity that ENSO explains the best. The statistical significance of TCI_opt_ argues for the importance of this unified approach, which is different from past approaches that deal separately with factors such as intensity, frequency, and TC activity. TCI_opt_ is the geometric version of prediction values from an Ordinary Least Squares regression model as

2 $${\rm{SOI}} \sim \,{\rm{INT}}\,+{\rm{FRQ}},$$

where “~” denotes a statistical relationship between the terms on the left- and right-hand sides. Then the angle 30.4° is understood as the optimal for determining the regression coefficients (weights) for both the INT and FRQ variables.

## Results

**Figure 4:**
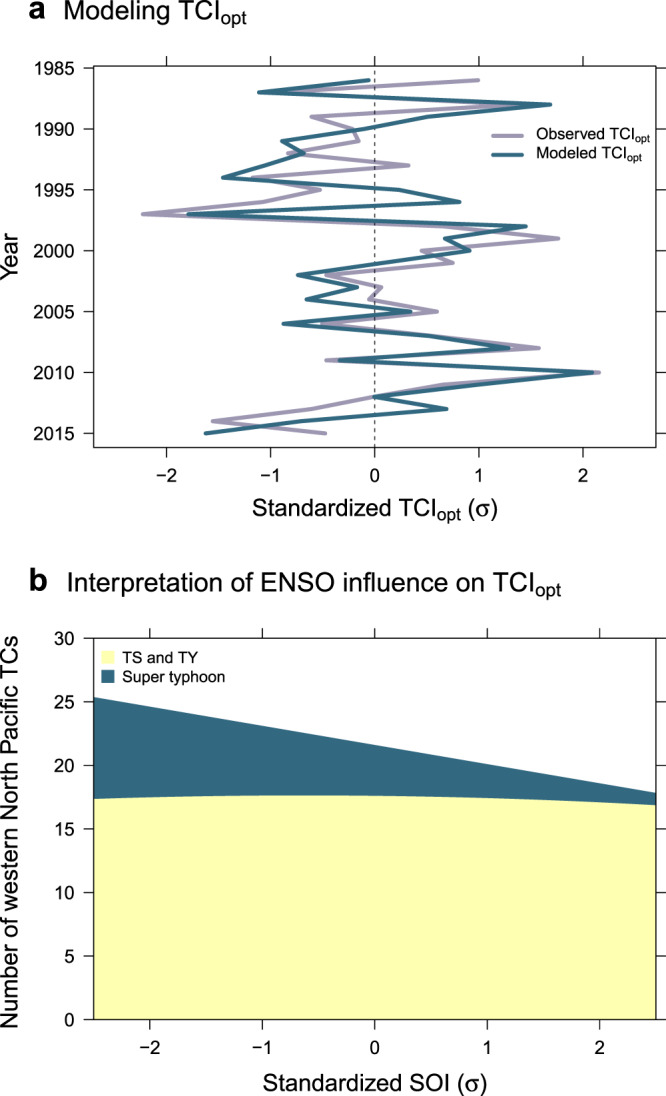
The quantified response of TC activity to ENSO. (**a**) Modeling TCI_opt_ by SOI, and (**b**) Interpretation of ENSO influence. From the regression of TCI_opt_ on SOI, the total number of WNP TCs and super typhoons (LMI ≥ 130 kt) are predicted over the range of SOI values.

The annual variation of TCI_opt_ and its modeled values by SOI are depicted in Fig. 4a. As the modeled TCI_opt_ is the regression onto SOI, the standardized values of the predictions (modeled values) have the same sign of the standardized SOI. Thus, the abscissa values in Fig. 4a,b represent the level of ENSO represented by standard deviations (σs). As noted earlier and as apparent in Fig. 4a, the annual standardized SOI fluctuates between −2σ and +2σ.

Using a unified analysis framework of TCI_opt_, this study shows how both INT and FRQ contribute to overall TC activity in response to ENSO through changes in the density distribution of genesis locations. The strength of this approach is that modeled TCI_opt_ interprets the INT and FRQ of TC activity together. The contributions of INT and FRQ to activity are quantified by the projections of the modeled TCI_opt_ onto INT and FRQ, as it were, TCI_opt_·cosθ and TCI_opt_·sinθ, respectively.

The resultant contribution of ENSO (indicated by SOI) to TC frequency (FRQ) alone is delineated in Fig. 4b. The FRQ of total WNP TCs from the modeled TCI_opt_ is seen as the straight outline to the upper side of the dark green area. The dark green area is the portion of super typhoons as defined as INT in Eq. (1). The number of total TCs and super typhoons are returned from the results (Table 1). The average number of super typhoons in association with ENSO is quantified as 7.1 out of 24.6 WNP TCs in the El Niño years at −2σ SOI level, while 1.5 out of 18.6 in the La Niña years at +2σ SOI. The difference of super typhoons between the two ENSO extremes is 5.6 (7.1–1.5). On the other hand, that the average number of non-super typhoon TCs stays at about 17.5 regardless of the ENSO state. Thus the average change of total TCs, 6.0 (24.6–18.6), can be considered as mostly contributed by super typhoons. This is the model output from the observed samples. The result is confirmed valid simply by the sample means between the warm and cold phases of ENSO (Supplementary Fig. S7). Other categories of JTWC’s typhoon classification such as ‘tropical storm’ and ‘typhoon’ are not separately examined in this study, but it is clear that the sum of these TCs contributes little to the total number of annual TCs. Overall, super typhoons are understood as the source of the higher (the lower) TC activity in the El Niño (La Niña) years. The same contribution of super typhoons is also found in a larger data period including more historical ENSO events (i.e., El Niño and La Niña events in 1970s and early1980s), while the values are considered less reliable due to the less accurate observations in the past^16,17^.

**Table 1:** Prediction from the regression of TCI_opt_ on SOI.

Standardized SOI (σ)	−2	−1	0	1	2
Total number of TCs	24.6	23.1	21.6	20.1	18.6
Number of super typhoons	7.1	5.5	4.0	2.7	1.5
Proportion of super typhoons	0.29	0.24	0.19	0.13	0.08

The results are deconvolved into the primary indicators of INT and FRQ, which are interpreted into annual WNP TCs and super typhoons.

The significance of the activity in response to ENSO is tested and confirmed by 95% confidence limits (see Fig. 3). The values in Table 1 are not individual predictions, but the interpretation of the activity on average. The spatial distribution of TC genesis locations at different ENSO status fails to fully explain these results.

## Summary and Discussion

This study quantifies the influence of ENSO on WNP TC activity and the contribution of super typhoons. It shows that SOI is a better index for representing the internal variation of ENSO compared to SST-based Niño indices when considering the impact ENSO has on typhoon activity. An optimal TC climate indicator defines the TC activity that ENSO explains the best. Modeled TC activity over the SOI range is effectively deconvolved into the contributions of overall TCs and super typhoons as follows:

- Historical levels of El Niño and La Niña span roughly between −2σ and +2σ of SOI.
- Over this SOI range, the average difference in total WNP TCs to El Niño and La Niña is 6.0 (24.6–18.6).
- The average difference in numbers of super typhoons is 5.6 (7.1–1.5), indicating that the response of WNP TC climate is mostly the result of more super typhoons.

The results also show that the number and genesis locations of non-super typhoons are little influenced by ENSO. The changes are mostly by the addition of super typhoons. The distribution pattern of TC genesis locations at different ENSO status fails to completely explain the results. The study is based on the assumption of a linear relationship between TC activity and ENSO. The linear perspective for this study is considered as simple and effective to catch the clear feature of ENSO’s influence on annual TC activity.

## Methods

This study employs the TC climate variability space developed by Kang and Elsner (2015). Space is made by a principal component analysis (PCA) using INT and FRQ as the two primary inputs. INT and FRQ are the vectors of annual TC intensity and frequency, respectively. The two principal components together with the two primary indicators form a continuous variability space (see www.nature.com/nclimate/journal/v5/n7/extref/nclimate2646-s1.pdf). Since all directional variabilities are continuously linked on the same space, the response of TC climate to its environment can be optimally detected (see Fig. 2), which enables the interpretation of different TC climate factors in a unified analysis framework.

The time period for a reliable consensus between observations from the Joint Typhoon Warning Center (JTWC) and the Japan Meteorological Agency (JMA) was suggested to be the years after 1984^17^. 1984 is the year when JMA employed Dvorak’s satellite analysis technique^18^ to the operational procedure. In this study, annual values over the 30 years (1986–2015) from June to November are used. The observational consensus of the LMI distribution by ENSO, between JTWC and JMA, is examined in the Supplementary Information (see Fig. S3), which supports the reliability of the study results produced by JTWC observations.

## Supplementary information

Supplementary Information

## Data Availability

SST-based ENSO indices such as Niño 1 + 2 (0°–10°S, 90°–80°W), Niño 3 (5°N–5°S, 150°–90°W), Niño 3.4 (5°N–5°S, 170°–120°W) and Niño 4 (5°N–5°S, 160°E–150°W) are extracted from the Extended Reconstructed Sea Surface Temperature (ERSST), version 4^19^. GMSST is also calculated from ERSST, version 4. SOI comes from the NOAA/Climate Prediction Center (CPC; http://www.cpc.ncep.noaa.gov/data/indices/soi). Best-track data from JTWC (http://www.usno.navy.mil/NOOC/nmfc-ph/RSS/jtwc/best_tracks) are used for TC climatology. All statistics are computed using R software (https://www.r-project.org), and the code is available online (http://www.rpubs.com/Namyoung/P2018a).
