# Supplementary material for: The contribution of super typhoons to tropical cyclone activity in response to ENSO
Source: Sci Rep. 2019 Mar 25;9:5046. doi: 10.1038/s41598-019-41561-y (PMC6434049; doi:10.1038/s41598-019-41561-y)
Supplement: Supplementary file 1 — Supplementary Information [file 41598_2019_41561_MOESM1_ESM.pdf]

Supplementary Information

The contribution of super typhoons to tropical cyclone activity in response to ENSO

Nam-Young Kang[1*], Dongjin Kim[1], and James B. Elsner[2]

[1]National Typhoon Center, Korea Meteorological Administration
[2]Department of Geography, Florida State University

1. Spatial distribution of super typhoons

Figure S1 shows the spatial distribution of super typhoons by (**a**) longitude and (**b**) latitude. In both distributions of **a** and **b**, super typhoons in the warm phase of ENSO (El Niño) are seen apparently larger than those in the cold phase of ENSO (La Niña). The number of super typhoons occurring to the west of 140°E decreases slightly within an El Niño environment, while the number apparently increasing to the east of 140°E. The increasing number of super typhoons also appears to the south of 20°N. Overall, the genesis location for super typhoons within El Niño environments moves toward the southeastern quadrant of the WNP. It is noted, however, that the feature seems mainly because of the increasing number of super typhoons in the southeastern part of the WNP rather than by a shift in the genesis locations.

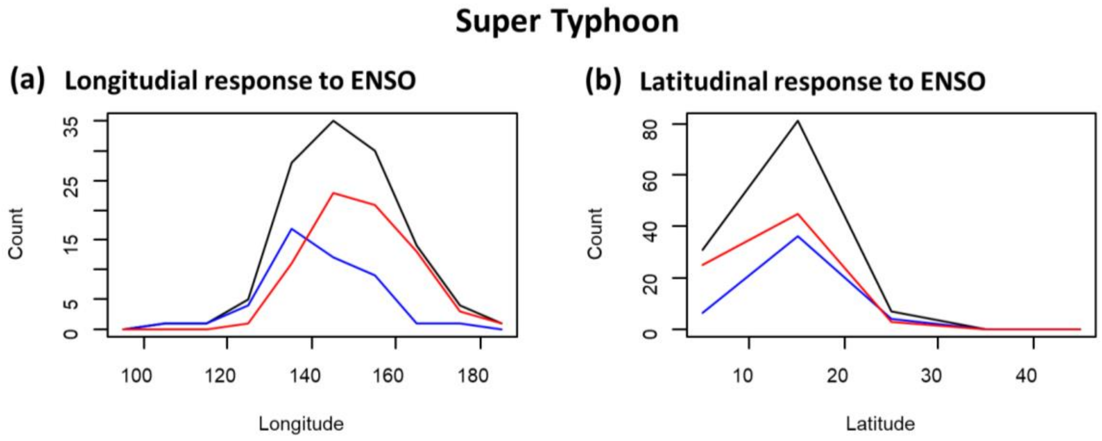

Figure S1. Spatial distribution of super typhoons. (a) Longitudinal, and (b) Latitudinal distributions in response to ENSO. Warm phase and cold phase of ENSO are colored in red and blue, respectively. The number of total TCs is shown in black line. Observations are for the months June through November (JJASON) over the 30-year period (1986–2015).

[1*]Corresponding e-mail: nkang.fsu@gmail.com

Figure S2 shows the spatial distribution of non-super typhoons by (**a**) longitude and (**b**) latitude. It is interesting that the difference in the numbers of non-super typhoons between the two phases of ENSO appears small in both **a** and **b**. Though some increase (decrease) in the southeastern (northwestern) part of WNP reflects the modulation of the genesis location, the amount looks small relative to the total and balanced (between the increase and the decrease). Considering Fig. S1 and S2 together, it is concluded that the distribution of the genesis location clearly changes but mostly by the addition of super-typhoons relative to non-super typhoons.

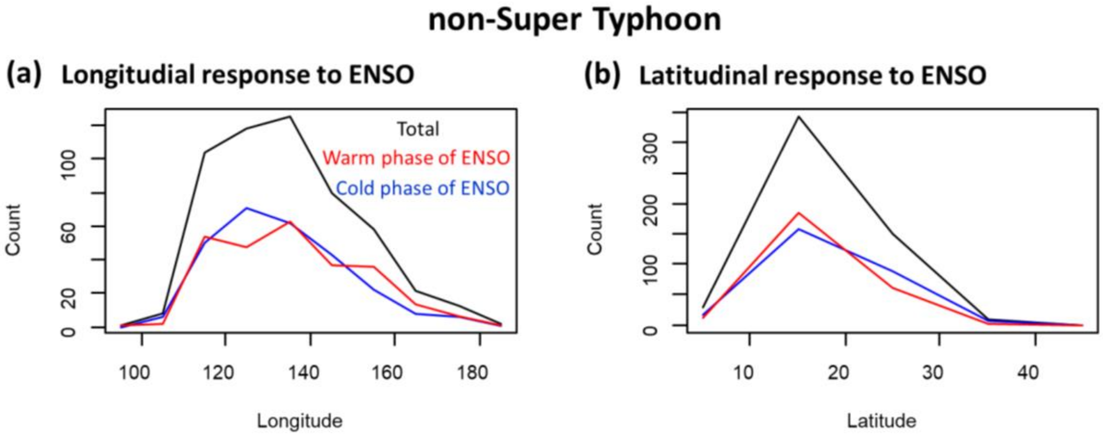

Figure S2. The same as in Fig. S1 except for non-super typhoons.

2. Density distribution of the LMIs over the SOI range

Figure S3 shows the density distribution of the LMIs over the SOI range. JTWC observations are compared to JMA observations. An observational consensus is confirmed by the similar patterns between them. The LMIs in JMA are the values equivalent to JTWC values. The values are estimated by the probability density function (PDF) having the same probability level as JTWC LMIs during JJASON over the 30-year period (2016– 2015). A vertical split around -0.3σ of SOI is seen spuriously made by fewer SOI samples (see Fig. S4). A horizontal split between the stronger and the weaker portions greater than 100 kt coincides with the findings in Lee *et al.* (2016). The stronger portion is considered as related to rapid intensification (RI). The number of RI-experiencing TCs and the warm phase of ENSO has a significant correlation (*r* = +0.45 [0.11, 0.70] 95 % CI), and all super typhoons except one during 1994 experienced RI during their lifetime. RI is considered as a background for super typhoons. This observation needs further investigation in a separate study. The number of super typhoons, on the other hand, are seen to have a stronger correlation with ENSO (*r* = +0.69 [0.44, 0.84] 95 % CI) than RI itself. Overall, ENSO's influence on LMIs is found to be most clear for the set of super typhoons.

Reference

Lee, C. Y., Tippett M. K., Sobel A. H., Camargo S. J, 2016: Rapid intensification and the bimodal distribution of tropical cyclone intensity. *Nature Communications*, **7**,10625.

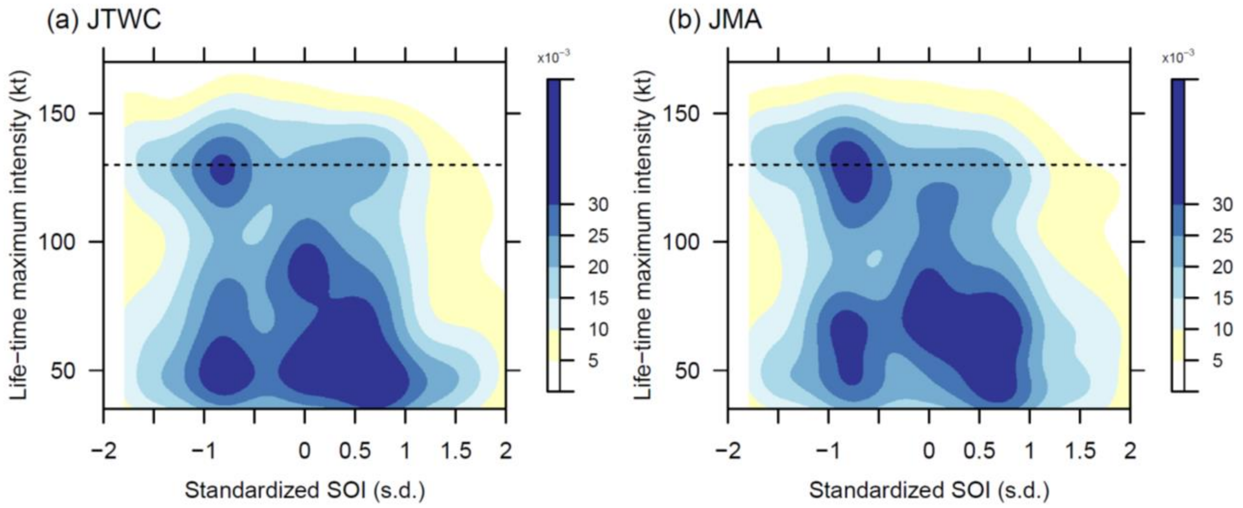

Figure S3. Density distribution of the LMIs over SOI. (a) JTWC and (**b**) JMA observations show similar patterns. LMIs for JMA are the equivalent to JTWC LMIs. Dashed line indicates the LMI of 130 kt, the threshold intensity for the category of super typhoons. Observations are for JJASON over the 30 years (1986–2015).

<u>**3. Logical structure of TC activity**</u>

Figure S4 shows the logical structure of TC activity. Here TC activity is understood as an indicator of TC activity including frequency and intensity. Annual TC frequency (FRQ) is the total number of TC occurrences. The intensity (INT) can be the annual mean LMI, quantile at a certain probability level, proportion (probability level) of a certain quantile, and so on. For example, the proportion of TCs with Saffir-Simpson's category 4-5 (CAT4·5) means the probability level of 113 kt.

The two time series show that the annual proportion of 113 kt and the annual quantile at the mean proportion (0.7) provide the same information, in principle. Once the proportion is taken into account for INT, the number of CAT4·5 can be understood as an indicator for TC activity, since both the INT and FRQ are involved in the quantity. This study defines INT as the annual proportion of TCs whose LMI exceeds 130 kt, that is the threshold of super typhoon category. Then, the number of super typhoons can be understood as indicating the TC activity, while it also contributes to INT and FRQ at the same time.

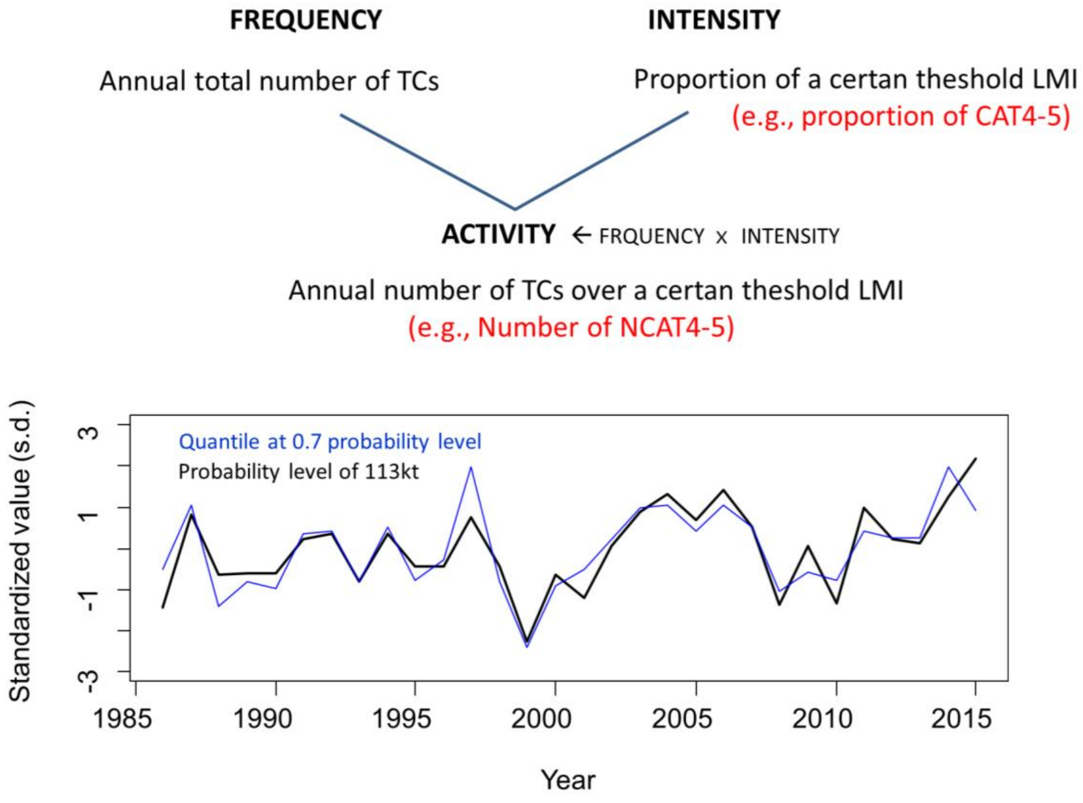

Figure S4. A diagram for the logical structure of TC activity. TC intensity can be indicated by the proportion of a certain threshold LMI. Annual values are averaged for JJASON.

4. Observed number of TC occurrences over the SOI range

Figure S5 shows the number of TCs over the range of SOI values. Each bar represents a year case. The total number of TCs includes the rest, and the number of RI-experiencing TCs includes super typhoons. Each regression line is added to the plot. The contribution of super typhoons is most clear to total TCs as well as RI-experiencing TCs. The regression lines show the classical way of modeling factors (black, red) individually, while Fig. 4 in the main manuscript is the overall interpretation of the single activity model (= TCI_{opt}) with FRQ (= total TCs) and INT (= proportion of super typhoons).

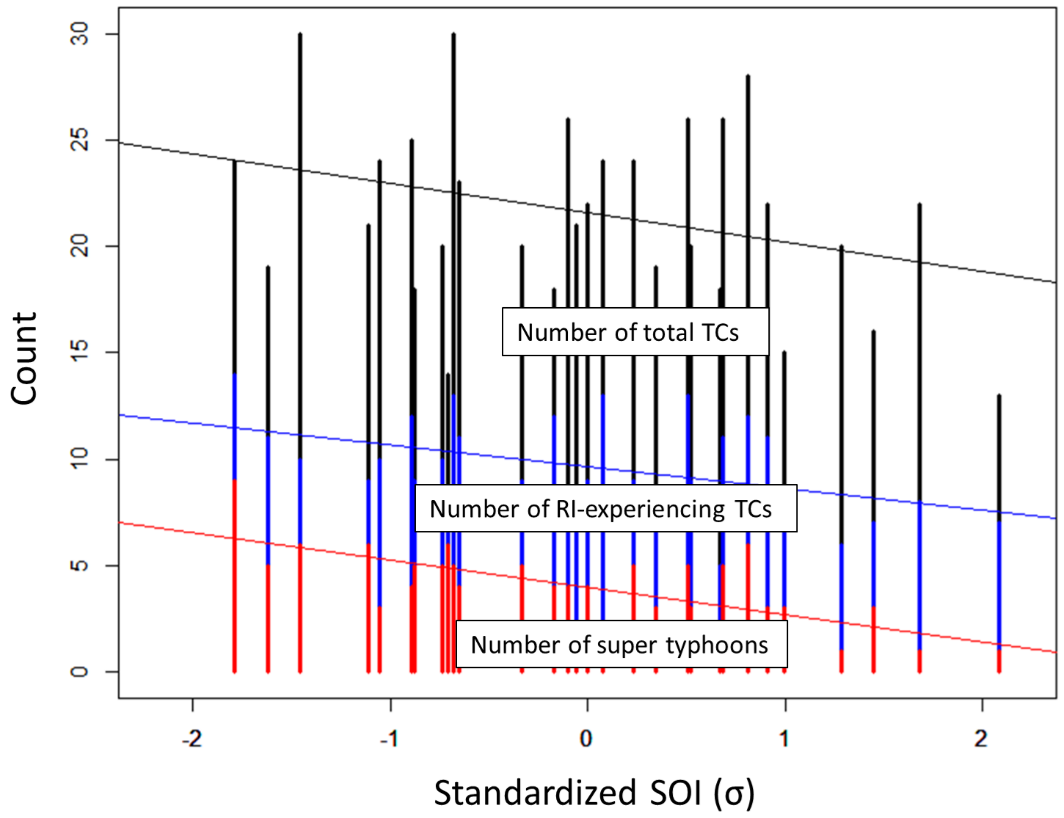

Figure S5. Number of TCs over the SOI range. Each bar represents a year case of total TCs (black), RI-experiencing TCs (blue), and super typhoons (red). The number of total TCs includes the rest, and the number of RI-experiencing TCs includes super typhoons. Each regression line is added to the plot. Observations are for JJASON over the 30 years (1986–2015).

5. Location of ACE and PDI in the TC climate variability space

Figure S6 shows the comparison of ACE (green line) and PDI (blue line) with TCI_{opt}. ACE and PDI as well as TCI_{opt} (orange line), are located around PC1 (the principal component of the in-phase relationship between INT and FRQ). The correlations of ACE (green line) and PDI (blue line) with the inverse sign of SOI (El Niño) are 0.68 and 0.69, respectively. The correlation between SOI and TCI_{opt} is 0.72.

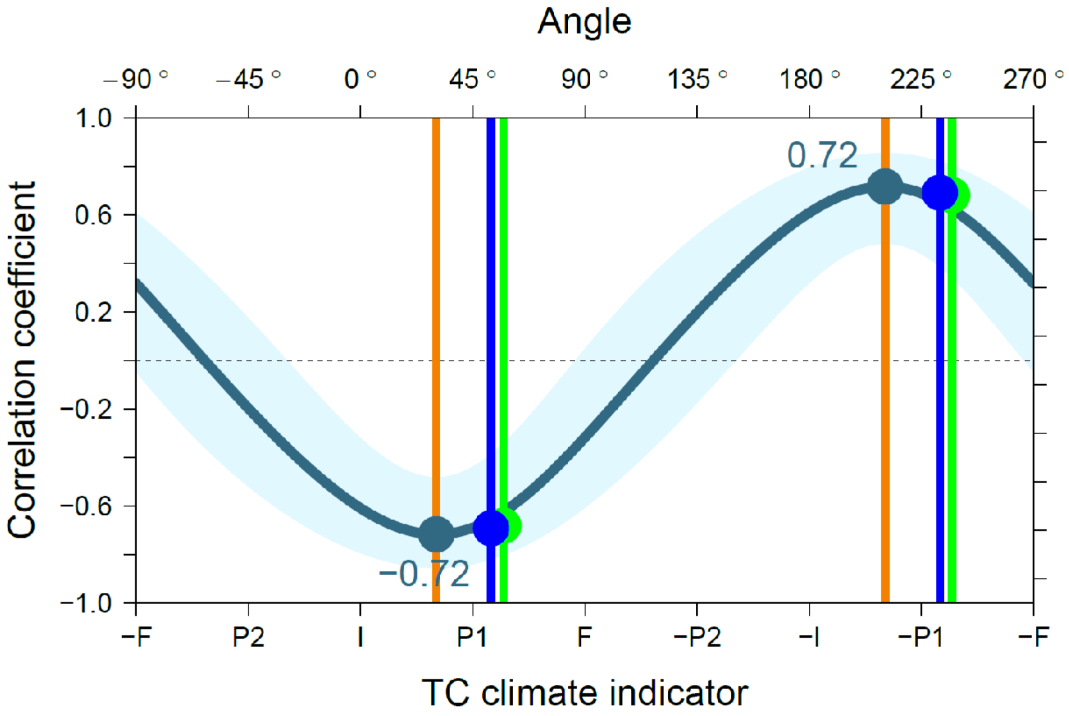

Figure S6. Comparison of ACE (green line) **and PDI** (blue line) **with TCI_{opt}** (orange line). Each indicates TC activity by construction. The correlations of ACE and PDI with the inverse sign of SOI (El Niño) appear 0.68 and 0.69, respectively. Annual values are averaged for JJASON over the 30 years (1986–2015).

6. Response of the number of observed TCs to ENSO status

Figure S7 confirms the model outputs by the sample means between the two different ENSO phases. The average of the annual non-super typhoon occurrences (blue bar) shows little difference (17.4 and 17.9) between warm and cold phases of ENSO, while the average of the annual super typhoon occurrences (blue bar) clear difference (4.8 and 3). Extreme two cases for each warm and cold phases of ENSO are averaged into 7 and 1, whose difference (6) is similar to the modeled difference in the main manuscript (see Table 1).

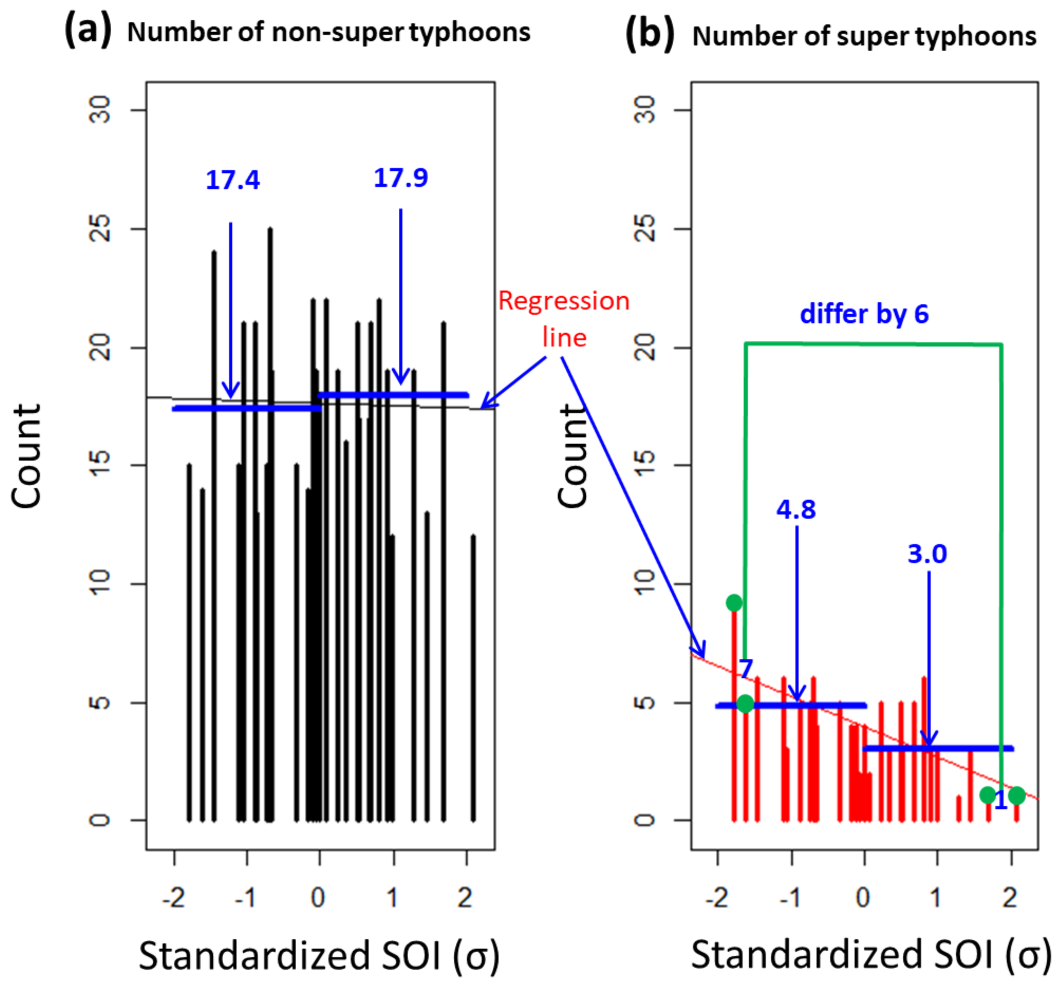

Figure S7. Response of the number of observed TCs to ENSO status. (a) Non-super typhoons, and (b) super typhoons show different responses to the level of ENSO status. Blue bars represent the average of annual number of TCs for each warm and cold phase of ENSO. Green dots show each two extreme samples in warm and cold phases of ENSO. Regression lines are added to the plots. Observations are for JJASON over the 30 years (1986–2015).
